# Fostering Local Ownership of Infection Prevention and Control Strategies: A Multi-country Program

**DOI:** 10.5334/aogh.5198

**Published:** 2026-04-20

**Authors:** Nicole C. McCann, Jeanette L. Kaiser, Nancy A. Scott, Tamara Hafner, Andre Zagorski, Mohan P. Joshi, Fozo Alombah, Allison Juntunen Morgan, José Antonio Requejo Domínguez, Veronika J. Wirtz

**Affiliations:** 1 Department of Health Law, Policy, and Management, Boston University School of Public Health, Boston, MA, United States; 2 Department of Global Health, Boston University School of Public Health, Boston, MA, United States; 3 USAID Medicines, Technologies, and Pharmaceutical Services (MTaPS) Program, Management Sciences for Health, Arlington, VA, United States

**Keywords:** antimicrobial resistance, COVID-19, community engagement, health systems, capacity strengthening, sustainability, sub-Saharan Africa, qualitative methods, mixed methods

## Abstract

*Background:* The United States Agency for International Development Medicines, Technologies, and Pharmaceutical Services (MTaPS) program supported partner countries to implement infection prevention and control (IPC)-related programs. We evaluated the extent to which MTaPS-supported IPC programs fostered local ownership, such that members of local or national-level health systems had agency in developing and running programs.

*Methods:* We surveyed three respondent groups involved in MTaPS IPC programs across eight African countries: (1) healthcare facility staff, (2) national-level stakeholders, and (3) MTaPS-contracted country implementers. Multiple-choice survey questions asked respondents to rate the quality of collaboration and capacity-building between their country and MTaPS, and the extent to which MTaPS fosters local ownership. Open-response questions inquired about factors that did or did not foster local ownership. We described the proportion of respondents reporting each multiple-choice response option, and conducted qualitative content analysis of open responses to generate themes about respondent perceptions of MTaPS support and local ownership.

*Results:* We included 85 survey respondents: health facility staff (56%), MTaPS-contracted country implementers (29%) and national-level stakeholders (14%). Nearly all respondents rated the quality of MTaPS collaboration and capacity building “good” or “excellent.” Overall, 75%–92% of respondents rated the quality of MTaPS collaboration and capacity strengthening as “mostly” supportive of local ownership and 8%–25% rated it “sometimes,” supportive (0% selected “rarely/never”). Qualitatively, respondents described six activities as conducive to local ownership, including training, data collection/monitoring, stakeholder engagement, guideline/protocol standardization, creation/development of local committees, and supervision/mentorship/direct technical assistance. A reported barrier to MTaPS’ support of local ownership was the inconsistent implementation of activities.

*Conclusions:* IPC programs should continue to prioritize strategies for fostering local ownership, particularly as the funding landscape shifts. To increase IPC program sustainability in advance of future infectious disease threats, additional resources are needed to scale up activities perceived as conducive to local ownership.

## Background

Infection prevention and control (IPC) is an evidence-based approach to protect patients and health workers from harm related to avoidable infections. According to the World Health Organization (WHO), effective IPC requires the continuous engagement of stakeholders at all levels of the health system, including policymakers, facility managers, health workers, and those who access health services [1]. As a result, it is essential for countries implementing IPC programs to prioritize local ownership, such that members of local- or national-level health systems have agency in designing and participating in IPC improvement measures and interventions [2].

From 2018 until its dissolution, the United States Agency for International Development (USAID) Medicines, Technologies, and Pharmaceutical Services (MTaPS) program supported USAID Global Health Security Agenda (GHSA) partner countries in antimicrobial resistance (AMR) containment. GHSA was a collaborative multisectoral global partnership supporting countries to enhance capacities to prevent, detect, and rapidly respond to infectious disease threats, and to implement the International Health Regulations (IHR) [2, 3]. MTaPS supported countries in the areas of multi-sectoral coordination on AMR (MSC-AMR), antimicrobial stewardship (AMS), and IPC. MTaPS began supporting 10 countries in these areas between September 2018 and March 2019, and an additional three countries in 2019–2020 [4]. In March 2020, when the COVID-19 pandemic escalated, MTaPS additionally began supporting a specific IPC strategy targeting the outbreak response of these and additional countries [4]. The goal of MTaPS’ support included the implementation of activities which promoted the WHO’s IPC core components for health facilities [5], including: (1) functional IPC programs, (2) national and facility-level IPC guidelines, (3) IPC education and training, (4) hospital acquired infection (HAI) surveillance, (5) use of multimodal strategies, (6) monitoring and feedback, (7) facility-level workload, staffing, and bed occupancy standardization, and (8) meeting built-environment standards at the facility level (e.g., water availability, cleaning, and waste practices). These activities ultimately aimed to prevent and control infectious diseases, to reduce AMR, to enhance pandemic preparedness, and to improve overall patient safety and quality of care.

Local ownership is a critically important principle and goal for development work. It is achieved through a sustained commitment to building the capacity of local actors via inclusive participatory processes, accompanied by international partners [6]. Local ownership requires long-term engagement and the empowerment of social and political actors to lead and implement initiatives. Local ownership is recognized according to the WHO Framework for Community Engagement as the highest level of engagement on an engagement spectrum and involves empowering communities to develop systems for self-governance and priority setting and to establish and manage the implementation and sustainability of interventions with external support [7, 8]. The MTaPS approach aligns with these principles.

The MTaPS approach prioritized fostering local ownership over AMR and IPC systems through consistent and continual collaboration between existing stakeholders at all levels, multisectoral coordination within and between existing legal and governance structures, capacity and systems strengthening initiatives, and technical support to allow stakeholders to design interventions which build on and strengthen existing IPC systems [2]. MTaPS aimed to develop trust from stakeholders through long-term collaboration with countries; being accessible to stakeholders from national, regional, and local levels of the health system; ensuring MSC-AMR and IPC initiatives implemented were highly relevant to local contexts; and advocating for integration of initiatives into all levels of governance.

The current analysis is part of a broader evaluation of the MTaPS program. Here, we use qualitative and quantitative survey data to assess stakeholder perspectives and experiences related to the extent to which MTaPS interventions fostered local ownership. This is the first study to evaluate whether the theory behind MTaPS’ approach to fostering local ownership translated into practice and is particularly relevant given the changing funding landscape. Evidence on local ownership can guide future decisions on program design.

## Methods

### Program description

**Figure 1:**
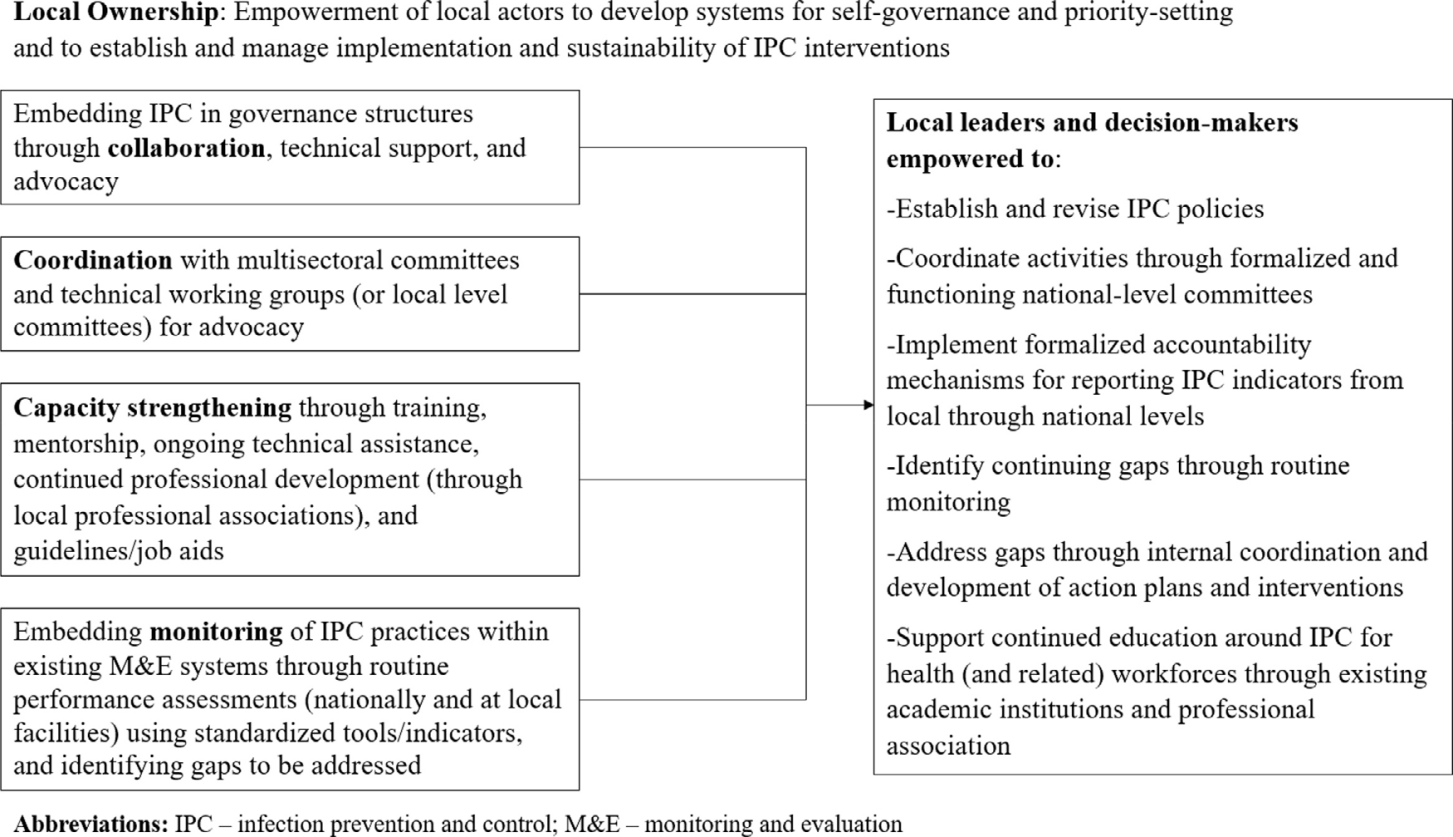
Simplified model of how MTaPS activities theoretically fostered local ownership.

The MTaPS program intervention package varied by country, and the level of intensity at the facility-level varied depending on need. The MTaPS interventions (Figure 1) included embedding IPC in governance structures through collaboration, technical support, and advocacy; coordination through existing multisectoral committees and technical working groups (or local-level committees); support to governance structures and formation/revisions of policies, frameworks, action plans, and guidelines; IPC program management and technical support at the national, subnational, and facility levels; institutional and human resource capacity strengthening for IPC through training, mentorship, ongoing technical assistance, continued professional development (through local academic and professional associations), and guidelines/job aids; and establishment and support of systems for IPC compliance monitoring and continuous quality improvement (e.g., through repeated use of WHO and national assessment tools). During COVID-19, IPC activities were expanded at all levels, with guidance specific to COVID-19 precautions for healthcare workers, patients, communities, and households.

Through participation in the MTaPS activities, national and health facility stakeholders were theoretically empowered to: ensure continued governance of IPC policies; coordinate activities at multiple levels; implement formal accountability mechanisms; identify and address gaps in IPC practices; and support continued education of relevant workforces (Figure 1).

A more detailed theory of change for MTaPS’ interventions is depicted in the program’s logic models (Figures S1 and S2). In the models, the activities affect short-, medium-, and long-term outcomes, including guideline compliance, routine implementation and monitoring of IPC activities, improved hospital outcomes (including reduced incidence of HAI, reduced lengths of stay, and reduced cost for hospital visits), and ongoing improvement of adherence to IPC standards.

### Study design

To understand stakeholder perspectives and experiences, we administered an online survey to respondents in the following eight African countries, which have received GHSA and COVID-19 IPC support from MTaPS: Cameroon, Côte d’Ivoire, Ethiopia, Kenya, Mali, Senegal, Tanzania, and Uganda. We captured respondents’ country of work, employment position, length of employment, and the MTaPS-supported activities which they reported participating in. The survey was written in English and was translated into French.

We sent the survey to health facility staff, national-level stakeholders (e.g., Ministry of Health officials involved in the program), and MTaPS-contracted country implementor staff in each country. The MTaPS-contracted country implementers were local health practitioners employed and trained by MTaPS to support the government and local facilities with technical assistance. The survey covered a range of questions related to respondents’ perceptions of the MTaPS program (Supplement, pages 2–17); for this analysis, we analyzed questions focused on local ownership. To assess the extent of local ownership, we asked respondents about the ways in which MTaPS engaged with national and facility-level staff regarding the quality of collaboration, coordination, and capacity strengthening activities. We also asked respondents to rate the supportiveness of MTaPS’ approaches for fostering local (in-country) ownership over IPC programs. Response options were on a four-point Likert scale from poor to excellent. The survey additionally asked several open-ended questions seeking more detailed information on the most important activities and how those activities fostered local ownership.

### Sampling and data management

We used a convenience sample of respondents engaged with the MTaPS-supported GHSA or COVID-19 IPC activities between 2019 and 2023. Surveys were self-administered through Boston University’s Qualtrics Research Suite [9]. MTaPS country leads disseminated links to the online surveys to eligible potential respondents through email and WhatsApp channels; respondents chose their preferred language (English or French). Open-ended responses for surveys conducted in French were translated using Google Translate [10]; translations were validated and corrected by a French-speaking member of the research team.

### Ethical considerations

Ethical approvals were obtained from the Boston University Medical Campus Institutional Review Board. The protocol was deemed exempt as nonhuman subjects research (Protocol #H-43699). Recruitment occurred between April 14, 2023 and April 30, 2023. Survey respondents read a brief description of the survey before deciding whether or not to participate, and gave informed consent before participation. Survey respondents were assured of their confidential responses.

### Analysis

We described the frequency of respondent characteristics and the proportion of respondents who participated in each type of MTaPS-supported activity. For multiple-choice questions, we calculated the proportion of respondents who selected each Likert scale response option.

For open-ended questions, qualitative responses were exported in Microsoft® Excel and analyzed using content analysis [11]. A codebook was developed inductively by two researchers on the study team for each question by reading through all responses, and each response was then reread and assigned relevant codes (up to four). Inductive codes were organized based on activities in the MTaPS program description and theory of change. Major themes were identified for each question based on the volume of each code. Here, we present key themes with illustrative quotes. Minor edits were made to some quotes for spelling or grammar.

## Results

### Survey respondent characteristics

Among the 85 survey respondents, the majority were health facility staff (56%), followed by MTaPS-contracted country implementers (29%), and national-level stakeholders (14%) (Table 1). Kenya and Cameroon were the most widely represented countries, comprising over half of the total sample, with 29 and 19 respondents, respectively. Approximately two-thirds of health facility staff and national-level stakeholders were the IPC focal points locally or nationally, or were members of an IPC or equivalent committee. Nearly half of the health facility staff were in clinical professions. MTaPS-contracted country implementers held various technical positions. The majority of all respondents had been in their positions between 1 and 5 years.

**Table 1:** Characteristics of survey respondents by respondent type (*n* = 85).

CHARACTERISTICS, *N* (%)	HEALTH FACILITY STAFF *N* = 48	NATIONAL-LEVEL STAKEHOLDERS *N* = 12	MTAPS-CONTRACTED COUNTRY IMPLEMENTERS *N* = 25
**County**			
Cameroon (*n* = 19)	13 (27)	3 (25)	3 (12)
Côte d’Ivoire (*n* = 9)	3 (6)	3 (25)	3 (12)
Ethiopia (*n* = 4)	1 (2)	1 (8)	2 (8)
Kenya (*n* = 29)	20 (42)	2 (17)	7 (28)
Mali (*n* = 8)	5 (10)	1 (8)	2 (8)
Senegal (*n* = 10)	6 (13)	2 (17)	2 (8)
Tanzania (*n* = 3)	0 (.)	0 (.)	3 (12)
Uganda (*n* = 3)	0 (.)	0 (.)	3 (12)
**Profession^a^**			
IPC focal point, on IPC committee, or equivalent	32 (67)	8 (67)	–
Clinical (e.g., medical doctor, clinical officer, nurse, pharmacist)	23 (48)	1 (8)	–
Nonclinical (e.g., public health officer, laboratory officer, microbiologist, sanitation engineer, surveillance)	5 (10)	3 (38)	–
Technical advisor/officer/assistant	–	–	21 (84)
Other	2 (4)	1 (8)	4 (16)
**Years in role**			
<1	3 (6)	0 (.)	5 (20)
1 to <5	23 (48)	7 (58)	19 (76)
5 to <10	11 (23)	4 (33)	0 (.)
10 or more	11 (23)	1 (8)	1 (4)

Abbreviations: IPC: infection prevention and control

^a^Some respondents reported both a medical profession and their position as an IPC focal point or involvement in the IPC committee, so columns sum to over 100%.

**Figure 2:**
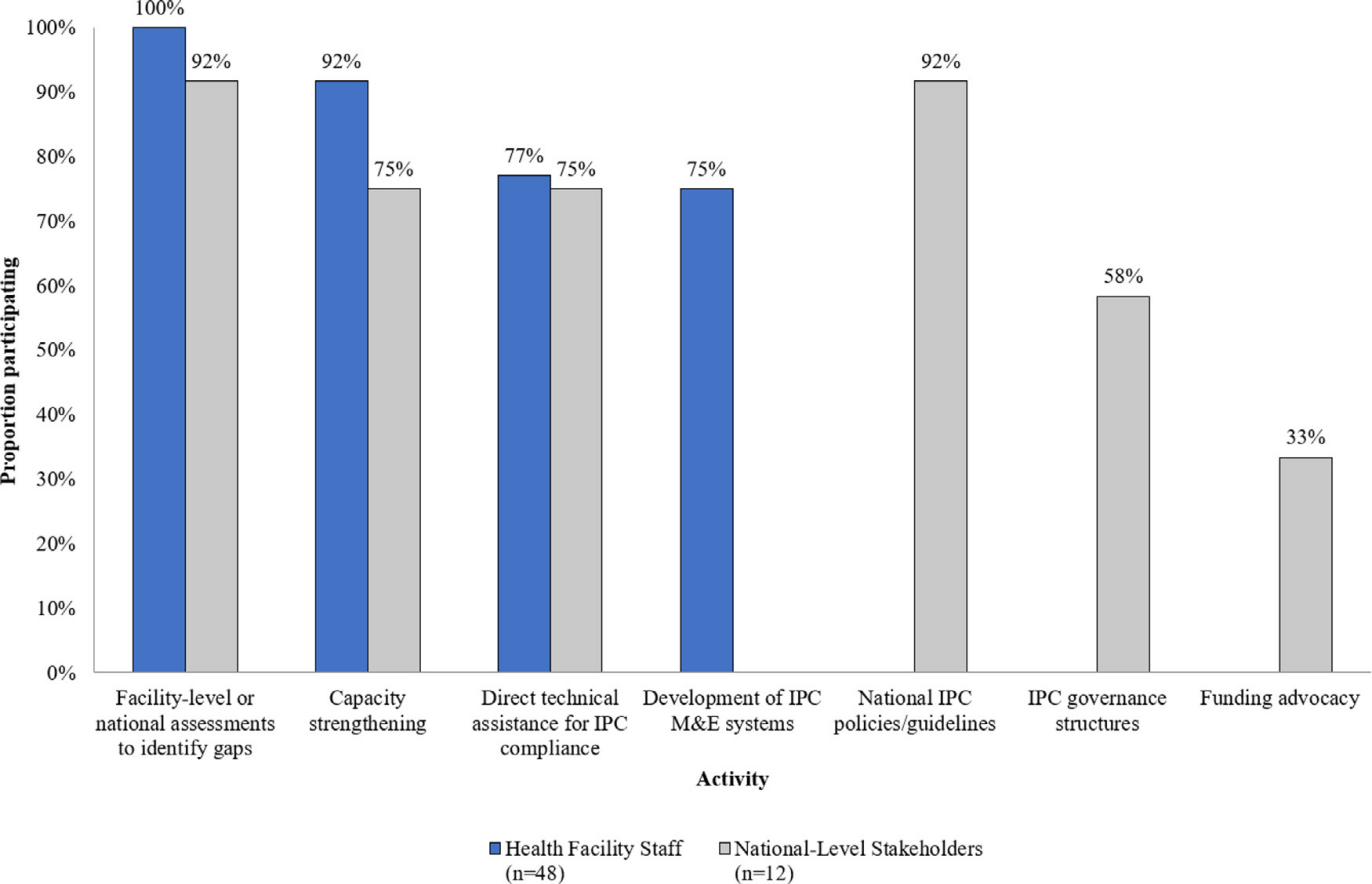
Reported MTaPS-supported activities survey respondents participated in^1^. Abbreviations: M&E: monitoring and evaluation ^1^Question not asked of MTaPS country staff.

Health facility staff and national-level stakeholders reported participating in a range of MTaPS-supported activities (Figure 2). Nearly all (92%) health facility staff and 75% of national-level stakeholders reported having participated in a form of capacity strengthening. While 85% of health facility staff reported participating in in-person trainings, fewer reported participating in other types of capacity strengthening activities, including in-service mentorship (33%), e-learning courses (38%), or micro-learning (15%) (data not shown). All health facility staff and nearly all (92%) national-level stakeholders participated in facility-level IPC assessments, and approximately 75% of each respondent group participated in direct technical assistance to health facilities. Three-quarters of health facility staff participated in the development of IPC monitoring and evaluation systems at their facilities. Nearly all (92%) national-level stakeholders participated in the development, updating, and/or dissemination of national IPC guidelines, the majority (58%) participated in IPC governance structures (i.e., in national or sub-national IPC technical working groups and/or worked to establish IPC committees at one or more health system levels), and 33% participated in advocacy for funding initiatives.

### Stakeholder perspectives on quality of MTaPS activities

**Figure 3:**
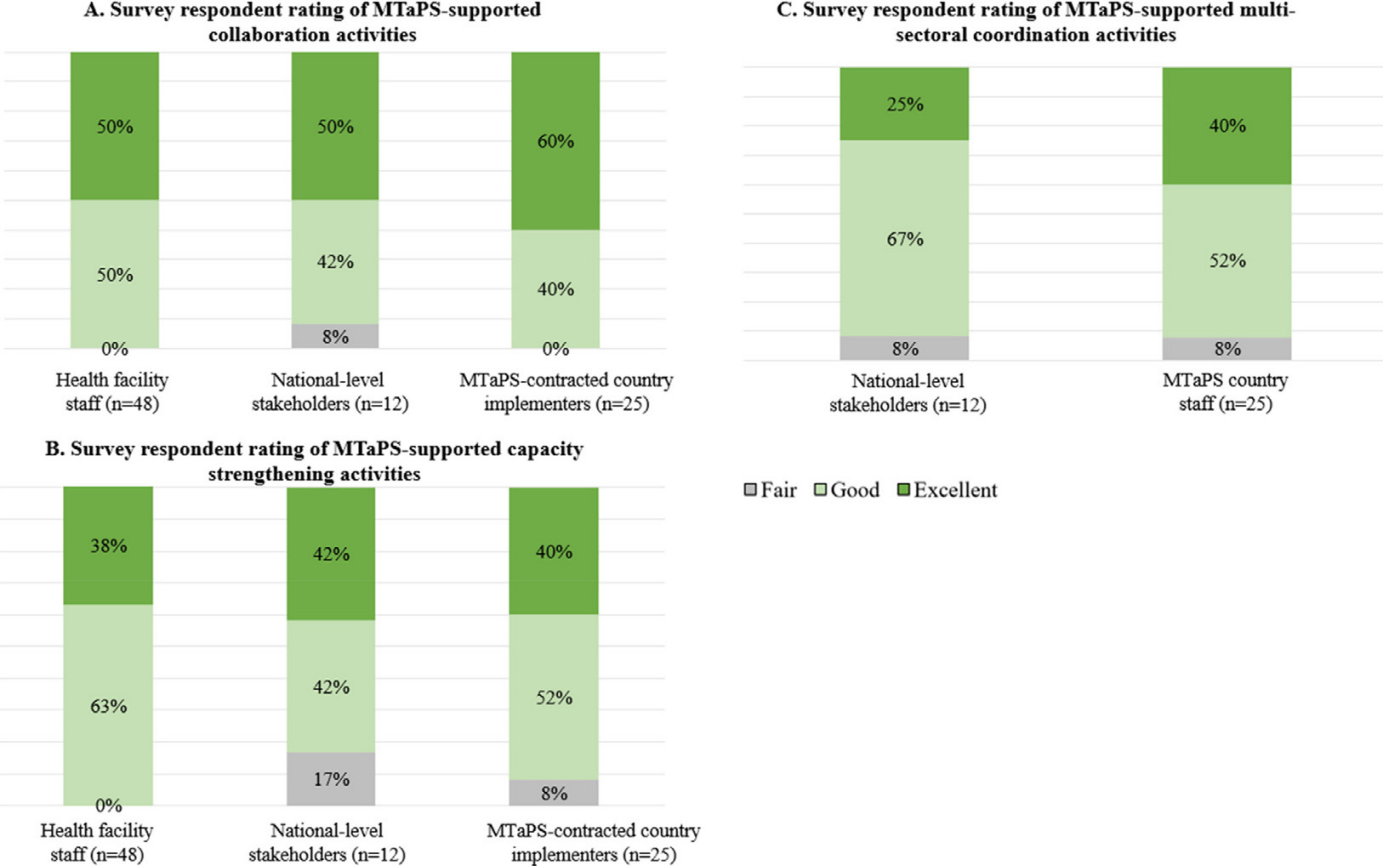
Survey respondent ratings of quality of MTaPS-supported **(A)** collaboration, **(B)** capacity strengthening and **(C)** multisectoral coordination activities^1^. ^1^No respondents selected the response option of “poor” for any of these questions.

Nearly all respondents in all groups rated the quality of MTaPS collaboration, multisectoral coordination, and capacity strengthening activities as “good” or “excellent” (Figure 3).

### Stakeholder perspectives on fostering local and national ownership

**Figure 4:**
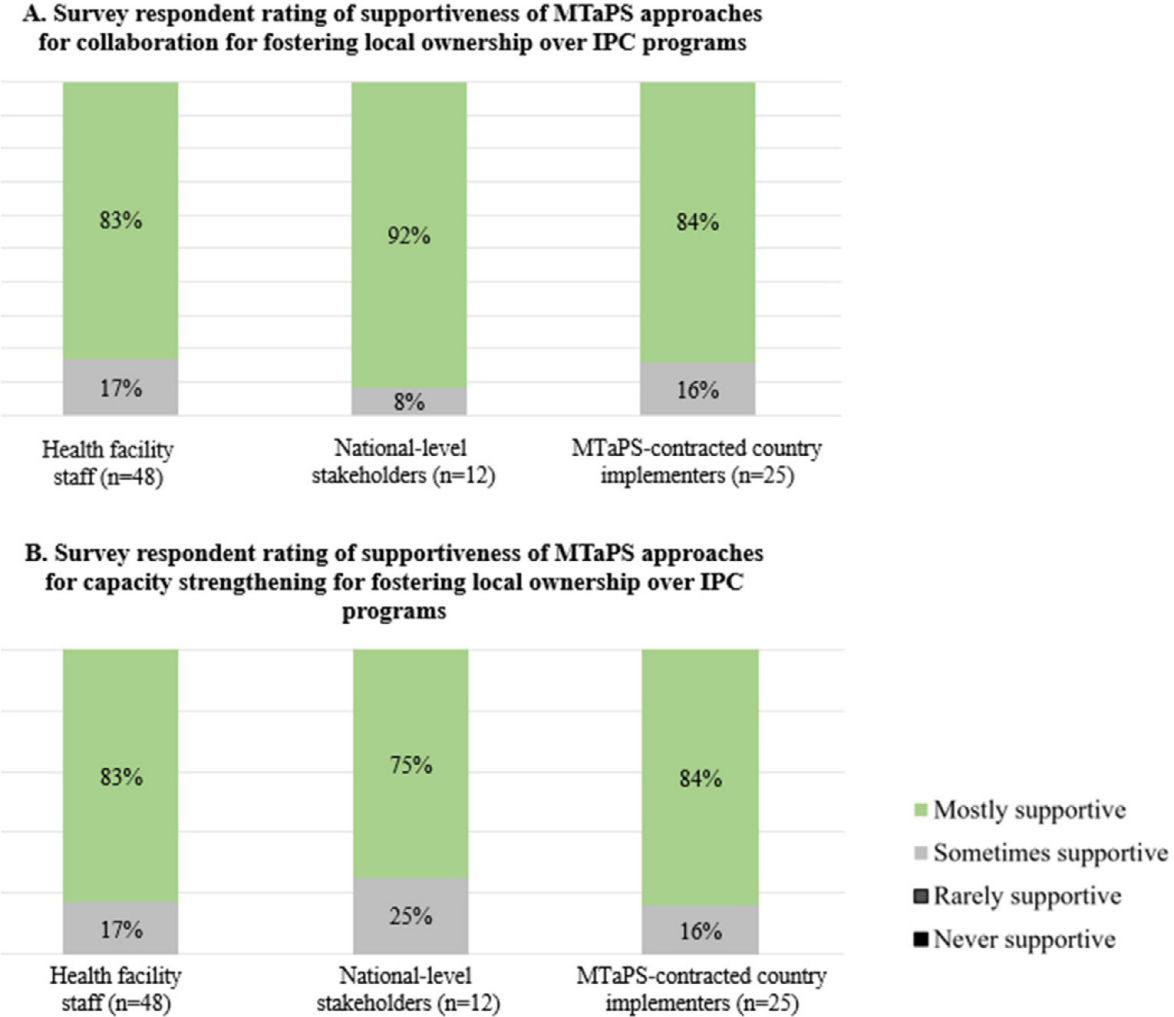
Survey respondent rating of supportiveness of MTaPS approaches for **(A)** collaboration and **(B)** capacity strengthening to foster local or national ownership over IPC. ^1^No respondents selected the response option of “rarely/never” for any of these questions. ^2^Question not asked for coordination activities.

Respondents perceived that MTaPS approaches for collaboration and capacity strengthening “mostly” or “sometimes” supported local ownership of IPC programs (Figure 4). Of the three stakeholder groups, national-level stakeholders most frequently reported that MTaPS’ collaboration approaches were “mostly” supportive of local ownership, and least frequently reported that capacity strengthening approaches were “mostly” supportive of local ownership. Health facility and MTaPS-contracted country implementers, 83% and 84% respectively, generally agreed that the collaboration and capacity strengthening approaches were “mostly” supportive of IPC programs’ local ownership.

Qualitatively, survey respondents described six overarching activities that they believed fostered local ownership of IPC systems: (1) training and capacity strengthening through in-person and virtual trainings; (2) collection and use of data for decision-making and quality improvement; (3) engagement with and coordination of stakeholders; (4) standardization of care through guidelines and protocol; (5) creation or strengthening of local committees; and (6) supervision, mentorship, and direct technical assistance by MTaPS. Each activity is explained further below, including illustrative quotes. Because no respondents selected “rarely” supportive of local ownership, no respondents were prompted with the opportunity to offer suggestions for improvement, specifically for fostering local ownership.

#### Activity 1: Training and capacity strengthening through in-person and virtual trainings

Many respondents from all groups primarily described the effects of in-person training, on-the-job training, e-learning courses, and “just-in-time” training as key to fostering local ownership (illustrative quotes shown in Table 2). National-level stakeholders highlighted the importance of MTaPS in developing training curricula and focused on the in-person training, which they perceived as most impactful. Some respondents specified that training of individuals at different levels (e.g., local) and in different positions (e.g., IPC focal persons, IPC committee members, and/or IPC coordinators) was critical. Some also noted that training of trainers was useful to reach more individuals. Some MTaPS-contracted country implementers and respondents noted the integration of IPC and MSC-AMR training into medical education as critical for long-term local ownership of the program in the future. A few respondents cited weaknesses in training activities, noting the irregularity of training, supervision, and monitoring activities; the low coverage of training, which only involved a few staff members per facility and not a wide range of worker cadres; and a lack of financial resources for adequate training. These comments tended to refer to earlier program years, pre-COVID-19.

**Table 2:** Illustrative quotes on how MTaPS support fostered local ownership through training and capacity strengthening activities.

RESPONDENT GROUP	ILLUSTRATIVE QUOTES
Health facility staff	“MTaPS helped the country by financing IPC just in time training, IPC full training and the realization of IPC assessment to identify gaps that were then addressed.”—Cameroon
National-level stakeholders	“[MTaPS activities which fostered national ownership included] training of national trainers; training of pool of regional trainers; training of staff in health establishments.”—Côte d’Ivoire
MTaPS-contracted country implementers	“[MTaPS activities which fostered national ownership included] updating the curriculum whereby the antimicrobial stewardship and IPC will be taught as standalone in the medical schools, and this will give more insight to the students before they start practicing.”—Uganda

#### Activity 2: Collection and use of data for decision-making and quality improvement

Many respondents from all three groups discussed the collection and use of data in various forms for monitoring, surveillance, evaluation, auditing, and behavior assessment (e.g., hand hygiene), patient outcomes (e.g., HAIs), or other aspects of IPC as key to fostering local ownership (Table 3). A few respondents described the general development of monitoring and evaluation systems as beneficial, as well as those systems being internal, with facility staff monitoring themselves. Respondents noted that data collected were frequently used to create action plans and develop efforts to achieve goals, leading to better performance, specifically regarding fewer HAIs among patients.

**Table 3:** Illustrative quotes on how MTaPS support fostered local ownership through the collection and use of data for decision-making and quality improvement.

RESPONDENT GROUP	ILLUSTRATIVE QUOTES
Health facility staff	“[Through the] baseline audit, followed by monthly auditing, we were able to know where we are as a facility in terms [of] hand hygiene compliance . . . [we] monitor our progress . . . as a facility we are able to implement strategies to prevent surgical site infections and monitor the outcomes.”—Kenya
National-level stakeholders	“[MTaPS activities which fostered national ownership included] evaluation of the capacities and functionality of IPC committees, development of an operational action plan in IPC for each health establishment, monitoring and support of the 20 health establishments in IPC supported by MTaPS.”—Côte d’Ivoire
MTaPS-contracted country implementers	“The following factors contributed to fostering ownership of IPC improvement activities at national level: (1) supporting the Ministry of Health to use evidence-based practice to improve the IPC program by using standardized tools, (2) Aligning USAID MTaPS IPC improvement plans with the Ministry of Health ‘s plan helps the Ministry of Health to sustain the IPC improvement efforts and achievements; (3) Supporting the Ministry of Health to develop national IPC monitoring tools, such as the Infection Prevention and Control Facility Level Assessment Tool [and] (4) Putting the Ministry of Health in the driver’s seat in all USAID MTaPS IPC improvement activities at sub-national and facility level.”—Ethiopia

#### Activity 3: Engagement with and coordination of stakeholders

National-level stakeholders and MTaPS-Contracted Country Implementors generally highlighted the importance of stakeholder engagement and coordination for fostering local ownership of IPC systems (Table 4). National-level stakeholders referenced their involvement throughout the various MTaPS activities and highlighted how the needs of the national party were incorporated into the MTaPS work plan. Both respondent groups highlighted the importance of national agency in their collaborative relationship with MTaPS, including the organization and activities of multisectoral task forces. MTaPS-Contracted Country Implementors described giving agency to national actors—particularly the Ministry of Health—to foster positive collaboration, the importance of consistency and flexibility of activities, and the incorporation of country-specific needs into the program. Several MTaPS-Contracted Country Implementors also discussed the importance of developing and maintaining relationships with diverse stakeholders across sectors, and across the multiple levels (i.e., national, sub-national, health facility). A few MTaPS-Contracted Country Implementors noted the critical role MTaPS played in bringing funding to the country for IPC, MSC-AMR, and COVID-19, and the program’s support for creating costed work plans and budgeting of activities.

**Table 4:** Illustrative quotes on how MTaPS support fostered local ownership through engagement with and coordination of local stakeholders.

RESPONDENT GROUP	ILLUSTRATIVE QUOTES
Health facility staff	No themes arose
National-level stakeholders	“Taking into account the needs of the national party in the MTaPS workplan”—Cameroon
MTaPS-contracted country implementers	“The collaboration with the Ministry of Health was impactful right from identifying the counties to be included (identifying the selection criteria-counties that had high infection rates among the population and health workers), revision of assessment tools, consensus from the council of governors, implementation and tracking compliance. Engagement with religious leaders and heads of learning institutions and capacity building for health workers working in prison facilities had great impact.”—Kenya

#### Activity 4: Standardization of care through guidelines and protocols

While many respondents from all groups mentioned guidelines as a key activity to foster local ownership, a few health facility staff explained that the guidelines, protocols, and standard operating procedures standardized care provided at the facility, describing this as beneficial (Table 5). A few national-level stakeholders also mentioned the development of the national strategic IPC plan as critical to providing the country with a unified direction and goal. MTaPS-Contracted Country Implementors explained that guiding documents and tools fostered national ownership as non-MTaPS-affiliated groups participated in developing and took ownership over their tools, including adapting them to address context-specific issues.

**Table 5:** Illustrative quotes on how MTaPS support fostered local ownership through the standardization of practices through guidelines and protocols.

RESPONDENT GROUP	ILLUSTRATIVE QUOTES
Health facility staff	“MTaPS helped the country and the Ministry of Health to elaborate the first IPC guidelines for healthcare facilities.”—Cameroon
National-level stakeholders	No themes arose
MTaPS-contracted country implementers	“[MTaPS activities which fostered national ownership included] support for the revision/development of national IPC guidelines under the leadership of the General Directorate of Health and Public Hygiene.”—Mali

#### Activity 5: Creation or strengthening of local committees

Multiple respondents mentioned the importance of MTaPS creating or strengthening local hospital committees on IPC, AMR, medicines and therapeutics, or hygiene (Table 6). A few respondents specified critical aspects of these committees, including functionality, regular meetings and report production, creation of objectives and action plans, and monitoring of activities within the health facility. Interestingly, when responding to questions about facilitators to local IPC ownership, national-level stakeholders did not frequently mention national-level IPC or AMR committees or technical working groups, but instead focused on lower-level committees, though this was not a strong theme.

**Table 6:** Illustrative quotes on how MTaPS support fostered local ownership through the creation and strengthening of local committees.

RESPONDENT GROUP	ILLUSTRATIVE QUOTES
Health facility staff	“MTaPS improved the IPC governance by strengthening the committee and ensuring the members understand their mandate. The committee activities improved the IPC practices in the facility.”—Kenya
National-level stakeholders	“MTaPs supported institutionalization of IPC governance in some counties.”—Kenya
MTaPS-contracted country implementers	“[MTaPS activities which fostered national ownership included] supporting the update of the Terms of Reference for the establishment of IPC committees, establishment of IPC committees in health establishments under the leadership of the General Directorate of Health and Public Hygiene.”—Mali

#### Activity 6: Supervision, mentorship, and direct technical assistance

Multiple respondents noted the importance of MTaPS supervision, mentorship, and direct technical assistance to strengthening the capacity of healthcare workers and fostering local ownership, though few provided details on the type or extent of these activities (Table 7). National-level stakeholders cited the importance of learning by demonstration and practice.

**Table 7:** Illustrative quotes on how MTaPS support fostered local ownership through supervision, mentorship, and technical assistance.

RESPONDENT GROUP	ILLUSTRATIVE QUOTES
Health facility staff	“Monitoring and supervision of work [was done] in a systematized way.”—Mali
National-level stakeholders	“[MTaPS activities which fostered national ownership included] supportive supervision; theoretical and practical in-person training on IPC; [and] technical supervision.”—Senegal
MTaPS-contracted country implementers	“We have created a community of practices among facilities, working and learning from each other to improve their IPC structures and practices. This has been possible because we have had several learning sessions where we brought together all the supported facilities.”—Uganda

For those who had less favorable perceptions about the degree of COVID-19-related multi-sectoral collaboration efforts, reasons included a lack of widespread implementation of collaboration activities, the need to involve more sectors, diversion of focus away from MSC-AMR and general IPC in favor of COVID-19, and a desire for more evaluations or situational analyses to pinpoint context-specific issues for targeting. For example, two respondents noted:

*“MSC activities were diverted towards addressing gaps in COVID-19, including guidance around antimicrobial use for suspected cases. IPC sits as a strategic objective in the National action plan and the county action plans on AMR and therefore implementation of those activities was prioritized, this increased stakeholder engagement toward addressing the pandemic. The monitoring of these interventions was not done and therefore cannot be rated as excellent.”*—Kenya, MTaPS-contracted country implementer

*“MTaPS supported the IPC hub meetings during which we had mainly stakeholders coming from the human health. Other sectors were rarely involved.”*—Cameroon, MTaPS-contracted country implementer

### Respondent perspectives on the effects of MTaPS support

Respondents widely agreed that these MTaPS-supported activities increased IPC governance, human resource capacity, and institutionalized good IPC practices at MTaPS-supported facilities. Health facility staff respondents specifically discussed improved IPC skills and practices in their health facilities and often related these to increased capacity of clinicians and allied staff through trainings, strengthened monitoring systems, and increased use of data for decision-making (identifying and addressing gaps), and established and connected governance systems to oversee IPC and ensure gaps were addressed. Some national-level stakeholders noted that increased awareness of IPC and the development of IPC as a technical field in and of itself helped contribute to the institutionalization of IPC practices. Some illustrative open-response quotes from national-level stakeholders and health facility staff respondents across five countries about the effects of MTaPS support are as follows:

“*The support of the MTaPS has impacted governance in terms of hygiene, management of diseases with epidemic potential, appropriate management and disposal of waste, acquisition of materials* and *medical equipment, [and] improvement of the working conditions of healthcare providers.”* —Côte d’Ivoire, health facility staff

*“MTaPS helped the country and the Ministry of Health to develop the first IPC guidelines for healthcare facilities. MTaPS helped the country by financing IPC just-in-time training, IPC full training and the realization of IPC assessment to identify gaps that were then addressed.”* —Cameroon, health facility staff

*“[MTaPS support resulted in an] active and well-organized IPC committee; improved waste management practices; positive behavior change among staff towards correct and consistent use of PPEs; [and] improved budgetary allocation for IPC projects.”*—Kenya, health facility staff

*“The MTaPS support has made a positive impact in the support of IPC activities because the support in training and monitoring and evaluation allowed us to correct many gaps in IPC.”—*Mali, health facility staff

*“The support of MTaPS has enabled us to strengthen our monitoring system in IPC, and AMR, the development of guidelines. [It increased] awareness on the importance of IPC and AMR. This support has also helped our health care facilities to effectively implement infection control programs in accordance with the guidelines on the main components of prevention and control programs infections by aiming to improve the quality and safety of health service delivery and health criteria of people who use these services.”—*Senegal, health facility staff

*“The support of MTaPS has made it possible to raise the IPC level of all these areas of intervention as well as the national level . . . IPC has become a technical field in Mali through the efforts of MTaPS.”*—Mali, national-level stakeholder

A few respondents connected improved IPC practices with better facility outcomes, including improved quality of patient care, improved hospital environments, and improved patient health outcomes, with fewer HAIs. Respondents described specific examples of improved IPC practices and their effects, such as shaving practices before caesarian sections, antibiotic prescribing practices, respiratory hygiene, and waste disposal:

*“We no longer shave all patients going to theater for caesarian section with blade as we used to do. If a mother has to be shaved, then it should be done in theater using calipers. Routine issuing of antibiotic to postnatal mother to prevent infection was stopped.”—*Kenya, health facility staff

*“*[MTaPS activities which fostered national ownership included] *harmonization of the prescription of antibiotics by clinicians.”* —Senegal, health facility staff

*“The support of MTaPS has greatly improved our practices. Especially in respiratory hygiene.”*—Senegal, health facility staff

*“The support of the MTaPS has impacted governance in terms of hygiene, management of diseases with epidemic potential, appropriate management and disposal of waste, acquisition of materials and medical equipment [and] improvement of the working conditions of healthcare providers.”*—Côte d’Ivoire, health facility staff

### Stakeholder perspectives on lessons learned

When asked about lessons learned, respondents discussed the importance of strong governance and coordination, including the roles of technical working groups, IPC committees, and focal persons to integrate local and national actions for IPC and MSC-AMR. Many also noted the importance of engagement and buy-in of stakeholders, particularly the diversity of stakeholders engaged. MTaPS-Contracted Country Implementors discussed the importance of facility ownership, in which facilities were leaders in establishing goals specific to their local contexts. Additionally, they noted the importance of facilities learning from each other. One participant noted the importance of awareness campaigns, such as the creation of World Hand Hygiene Day. Another respondent highlighted the importance of stakeholder engagement and ownership:

*“Engage all stakeholders at the start of the program, this is even better than handing the a perfectly crafted work plan; routine touch base is key”* —Uganda, MTaPS-contracted country implementor

## Discussion

We used an online survey with categorical and open response questions to assess the perspectives of health facility staff, national-level stakeholders, and MTaPS-contracted country implementers on the extent to which MTaPS’ IPC programs fostered local ownership of IPC interventions. Overall, respondents generally viewed MTaPS’ program positively and believed that program activities fostered local ownership. Broadly, respondents agreed that MTaPS-supported activities had positive effects, such as improving IPC governance, human resource capacity, and institutionalizing good IPC practices at facilities. Health facility staff particularly noted improved IPC skills and practices in their health facilities, resulting in better patient care and reduced HAIs. In open response questions, responses highlighted overarching activities that respondents believed helped to support local ownership of the program. Our findings suggest that prioritizing strategies for local ownership can effectively increase engagement and agency among health facility staff and national-level stakeholders, which has important implications for ensuring sustainability of programs over time [12, 13].

We found that the activities which respondents cited as key to fostering local ownership of IPC programs matched the MTaPS program description and theory of change. The six activities which were most often described by respondents as efforts that supported local ownership were: (1) training and capacity building, (2) data collection for decision-making and quality improvement, (3) stakeholder engagement, (4) standardization of care through guidelines and protocol, (5) creation/strengthening of local committees, and (6) supervision, mentorship, and direct technical assistance. Each of these six parameters can be mapped to an activity outlined in the MTaPS program logic models. Notably, these six activities, which were commonly discussed in open response questions, were consistent with the activities which respondents reported participating in, highlighting both the positive perceptions of such participatory activities as well as high fidelity to program design. These activities also reflected MTaPS’ priorities of fostering ownership and country self-reliance. For example, training and capacity building was one of the most frequently mentioned activities that fostered ownership, consistent with the guiding principle, which suggests that building and strengthening capacity of local organizations and providing technical leadership should be prioritized. Importantly, health facility staff respondents noted the importance of training staff at all levels, consistent with the systems strengthening approach that targets actors in several key areas. Also consistent with a systems-strengthening approach, respondents noted the importance of engaging diverse stakeholders generally by considering both national and local perspectives and needs. For example, the creation and strengthening of IPC committees to institutionalize IPC practices, including guidelines, data monitoring, and action plans, was perceived by respondents as a facilitator of local ownership.

In some cases, respondents noted areas that needed improvement for collaboration or capacity strengthening. For example, one respondent noted that during COVID-19, implementation of activities was prioritized, but because resource constraints, data monitoring was limited. Another respondent noted that most stakeholders involved in the MTaPS program were from the human health sector in their country, a point which highlights the need for and value of including diverse stakeholders. Some of the respondents noted barriers to local ownership, such as the lack of funding, inconsistency, and low coverage of training activities. In summary, respondents seemed to agree regarding the effectiveness of MTaPS’ approach and specific activities for fostering local ownership; however, in some contexts, likely due to limited resources, the activities were not optimally implemented, which might have limited success.

The importance of local ownership of interventions in low- and middle-income countries has been highlighted in prior work. For example, in Sierra Leone, a strengthened program was needed to address inadequate IPC measures, which contributed to high rates of Ebola from 2014 to 2016; as this program was implemented, ownership and leadership from the national Ministry of Health and Sanitation were found to be critical to the intervention’s success [14]. Additionally, interviews from key informants in Nigeria, Tanzania, and Ethiopia revealed that engaging multiple stakeholders in a national plan was the preferred approach for health priority setting [15]. A 2018 analysis discussed common challenges for implementing IPC programs in resource-limited settings, including limited resources and funding, inadequate infrastructure, and staff shortages. This analysis suggests several approaches that can counter these challenges, including approaches that increase local ownership such as engagement of leadership at all levels (national, facility, and department), and engagement of health workers [16]. Our survey-based analysis adds to this literature, suggesting that MTaPS’ aims and implementation strategies generally led to positive perceptions among a range of stakeholders who reported that local ownership of the program was strong.

The MTaPS program may have promoted facilitators to local ownership, which were not explicitly detailed in MTaPS’ priorities. A study using interviews from key informants in the United States and Zimbabwe investigated challenges, successes, and lessons learned during a transition period in which services were transferred from management by a US-based organization to a Zimbabwean organization [13]. The study analyzed interviews to generate recommendations for a successful transition to local ownership. Recommendations included developing a clear vision, empowering leadership, and engaging and mobilizing staff by providing infrastructure and technical assistance [13]. Our results are consistent with these recommendations, as respondents reported that engagement of stakeholders at all levels, including leaders within the Ministry of Health and at facilities, as well as staff training and technical assistance, promoted ownership.

Our analysis has several strengths. First, it utilized a survey with categorical and open response questions, and we were able to obtain a range of data, including overall and stakeholder perceptions as well as in-depth explanations of such perceptions. Additionally, the survey sample was relatively robust with national-level, facility, and MTaPS-contracted country implementors and respondents representing eight countries working with MTaPS. Different respondent types allowed us to triangulate themes between respondent groups who had different types of experiences with MTaPS. Lastly, as prior work has recommended that program implementers communicate with stakeholders to better understand perceptions and barriers of programs in local contexts [13], the contents of this survey-based analysis can serve as a method of dissemination of stakeholders’ perceptions of the program and, as such, contribute to addressing this recommendation.

The analysis also has several limitations. We recruited a convenience sample, representing individuals who had the availability to take the survey. While respondents represented eight countries, we did not get responses from all types of respondents (i.e., facility, national, and MTaPS) for all eight countries, and some countries had more responses than others. Because of small samples when stratifying by country, we were unable to analyze themes according to country, and we may have missed context-specific nuances. Additionally, such a sample may be subject to bias, including selection bias (e.g., those choosing to take the survey had different opinions than those choosing not to). Responses may also be subject to social desirability bias, although we aimed to mitigate this by only allowing access to full survey responses and identifying information to the third-party Boston University team, and de-identifying responses before sharing with MTaPS-associated authors. Finally, although we included qualitative analysis of open response question responses, we were not able to obtain the level of detail that may have been obtained through in-depth interviews, including variation in perceptions between respondent types (e.g., national-level stakeholders vs. MTaPS-contracted country implementors). Future research should investigate IPC program activities and their implementation processes in more depth to understand these dynamics, as well as how specific features impacted local ownership. For example, national-level stakeholders indicated a stronger preference for in-person training compared with facility-level staff. While some work has been done regarding the feasibility of virtual IPC training [17], additional evaluation comparing in-person and virtual options could be useful. Broadly, additional in-depth evaluation work in this space could be used to validate the findings in this paper and to inform future programming. Understanding the most efficient methods for fostering local ownership is increasingly important in light of recent scale-back of international aid and shifting funding environment.

In conclusion, our survey analysis found that among health facility staff, national-level stakeholders, and MTaPS-contracted country implementers involved in MTaPS’ IPC program, perceptions of how the program fostered local ownership were strongly positive. For respondents who reported gaps to collaboration or capacity strengthening, they were largely related to inadequate levels of implementation of program activities, partly due to limited resources; no respondents reported believing that activities hindered local ownership. These findings suggest that IPC programs may benefit from continuing to follow guiding principles for fostering local ownership, and that additional efforts and resources are needed to scale-up activities which are viewed positively by our sampled stakeholders in order to enhance the sustainability of programs over time in preparation for future infectious disease threats.

## Data Availability

The datasets generated and/or analyzed during the current study are not publicly available due to respondent privacy concerns, but are available from the corresponding author on reasonable request.

## Additional File

The additional file for this article can be found as follows:

Supplementary Appendix 1. From theory to practice: fostering local ownership of infection prevention and control strategies in a multi-country program.

## References

[r1] World Health Organization. Infection prevention and control [Internet]. n.d. Accessed April 10, 2024, https://www.who.int/health-topics/infection-prevention-and-control#tab=tab_1.

[r2] Joshi MP, Hafner T, Twesigye G, et al. Strengthening multisectoral coordination on antimicrobial resistance: A landscape analysis of efforts in 11 countries. J Pharm Policy Pract. 2021;14(1):27. 33648589 10.1186/s40545-021-00309-8 PMC7917520

[r3] World Health Organization. International health regulations [Internet]. n.d. Accessed April 11, 2024, https://www.who.int/health-topics/international-health-regulations#tab=tab_1.

[r4] Joshi MP, Alombah F, Konduri N, et al. Moving from assessments to implementation: Promising practices for strengthening multisectoral antimicrobial resistance containment capacity. One Health Outlook. 2023;5(1):7. 37055845 10.1186/s42522-023-00081-6 PMC10101730

[r5] World Health Organization. Infection prevention and control: Core components for IPC [Internet]. n.d. Accessed April 11, 2024. https://www.who.int/teams/integrated-health-services/infection-prevention-control/core-components.

[r6] Interpeace. Local leadership to local ownership—An essential element for effective peacebuilding and conflict prevention. Published 2018. Accessed January 26, 2025. https://www.interpeace.org/wp-content/uploads/2020/03/Why_Local_ownership_matters-policy_note-21-Sept.pdf.

[r7] World Health Organization. Community engagement: A health promotion guide for universal health coverage in the hands of the people [Internet]. Published 2020. Accessed April 10, 2024. https://www.who.int/publications/i/item/9789240010529.

[r8] World Health Organization. WHO community engagement framework for quality, people-centred and resilient health services. Published 2017. Accessed August 8,2024. https://www.who.int/publications/i/item/WHO-HIS-SDS-2017.15.

[r9] Qualtrics Research Suite [Internet]. n.d. Accessed April 10, 2024. https://www.bu.edu/tech/services/cccs/desktop/distribution/qualtrics/.

[r10] Google Translate. Google LLC. n.d. Accessed April 10, 2024, https://translate.google.com/.

[r11] Columbia Mailman School of Public Health. Content Analysis. n.d. Accessed April 10, 2024. https://www.publichealth.columbia.edu/research/population-health-methods/content-analysis#:~:text=Courses-,Overview,words%2C%20themes%2C%20or%20concepts.

[r12] Adams F, Zimmerman PA, Sparke VL, Mason M. Towards a framework for a collaborative support model to assist infection prevention and control programmes in low- and middle-income countries: A scoping review. Int J Integr Care. 2023;19:21851.

[r13] Vu M, Holec M, Levine R, et al. Working toward sustainability: Transitioning HIV programs from a USA-based organization to a local partner in Zimbabwe. PLoS ONE. 2022;17(11):e0276849. 36355839 10.1371/journal.pone.0276849 PMC9648773

[r14] Kanu H, Wilson K, Sesay-Kamara N, et al. Creation of a national infection prevention and control programme in Sierra Leone, 2015. BMJ Glob Health. 2019;4(3):e001504. 10.1136/bmjgh-2019-001504 PMC6570974 31263590

[r15] Kwete XJ, Berhane Y, Mwanyika-Sando M, et al. Health priority-setting for official development assistance in low-income and middle-income countries: A best fit framework synthesis study with primary data from Ethiopia, Nigeria and Tanzania. BMC Public Health. 2021;21(1):2138. 34801001 10.1186/s12889-021-12205-6 PMC8605935

[r16] Manchanda V, Suman U, Singh N. Implementing infection prevention and control programs when resources are limited. Curr Treat Options Infect Dis. 2018;10(1):28–39.

[r17] Kessy SJ, Gon G, Alimi Y, et al. Training a continent: A process evaluation of virtual training on infection prevention and control in Africa during COVID-19. Glob Health Sci Pract. 2023;11(2):e2200051. 37116932 10.9745/GHSP-D-22-00051 PMC10141425

