# Supplementary material for: Fostering Local Ownership of Infection Prevention and Control Strategies: A Multi-country Program
Source: Ann Glob Health. 2026 Apr 20;92(1):40. doi: 10.5334/aogh.5198 (PMC13109913; doi:10.5334/aogh.5198)
Supplement: Supplementary Appendix 1. — From theory to practice: fostering local ownership of infection prevention and control strategies in a multi-country program. [file agh-92-1-5198-s1.pdf]

Supplemental appendix: From theory to practice: fostering local ownership of infection prevention and control strategies in a multi-country program

Table of contents **Page number**

Survey questions 2-17

Figure S1: MTaPS logic model: MSC-AMR-related activities and outputs 18

Figure S2: MTaPS logic model: IPC-related activities and outputs 19-20

MTaPS evaluation survey administered to health facility staff, national-level stakeholders, and MTaPS-contracted country implementers

In the three sections below, we provide the surveys administered to health facility staff, national-level stakeholders, and MTaPS-contracted country implementers respectively. The questions analyzed in this analysis specifically are shown in red text.

1. ***MTaPS Facility Survey***

Introduction and consent

Hello. You are being asked to kindly participate in this survey. This survey is commissioned by MSH-led USAID Medicines, Technologies, and Pharmaceutical Services (MTaPS) program and conducted by researchers at the Boston University School of Public Health. You have been identified because you are a key stakeholder in one or more of the programs supported by MTaPS that focuses on infection prevention control, antimicrobial resistance stewardship, or multisectoral collaboration on antimicrobial resistance (AMR) in your country. We believe you have valuable experience with and/or knowledge of USAID MTaPS-supported IPC activities in your facility before and during the COVID-19 pandemic.

We want to understand the collective impact of these programs and to generate lessons learned for pandemic preparedness. Could you please fill out this survey now? It should take about 15-20 minutes. The information you provide will have no impact on you and will be kept confidential. We will store your information in ways we think are secure. We will store electronic files in computer systems with password protection and encryption. However, we cannot guarantee complete confidentiality. Results will only be presented in aggregate. Completely anonymized data may be posted to a repository. You will not receive any direct benefits or compensation; results will help to make future programs better.

At the end of the survey, we will ask if we can follow up if we have additional questions. If you choose, you can input your contact information. If you have any questions about this survey, please contact [Boston University personnel, redacted]

Do you agree to participate?

a. No
b. Yes

If respondent answers yes, the survey continues (if no, it ends):

Welcome note

Thank you for agreeing to participate. Your responses will help us to understand strengths and weaknesses of the MTaPS capacity strengthening approach and make improvements to these programs in the future. Please answer to the best of your ability. We greatly appreciate your participation and perspectives.

Section 1. MTaPS-supported programs and activities

1. Which MTaPS-supported programs supported your health facility <u>before</u> the COVID-19 pandemic? Select all that apply.
    a. GHSA AMR package: Multisectoral and partner coordination for AMR and IPC
    b. GHSA AMR package: IPC strengthening at the national or sub-national level
    c. GHSA AMR package: IPC strengthening at the facility level

2. Which MTaPS-supported programs supported your health facility during the COVID-19 pandemic? Select all that apply.
    a. GHSA AMR package: Multisectoral and partner coordination for AMR and IPC partner coordination for AMR
    b. GHSA AMR package: IPC strengthening at the national or sub-national level
    c. GHSA AMR package: IPC strengthening at the facility level
    d. MTaPS COVID-19 IPC package: Multisectoral and partner coordination for COVID-19 IPC at the national or subnational level
    e. MTaPS COVID-19 IPC package: COVID-19 IPC strengthening at the facility level

3. In what kinds of MTaPS-supported IPC activities have you been involved? Select all that apply.
    a. In-person trainings
    b. In-service mentorship
    c. E-learning (formal e-courses, use of social media platforms to deliver content, etc.)
    d. Micro-learning
    e. Health facility IPC assessments
    f. Direct technical assistance for compliance with IPC standards and guidelines
    g. Development of IPC monitoring and evaluation systems
    h. Other, please specify ___________

Section 2. Approaches to MTaPS collaboration with health facility stakeholders for Infection Prevention and Control (IPC)

4. Through what methods has MTaPS collaborated with your health facility? Select all that apply.
    a. Capacity strengthening of healthcare providers (trainings, mentorship, e-Learning)
    b. Development and dissemination of IPC guidelines and toolkits
    c. Establishment or strengthening of the facility IPC committee
    d. Health facility IPC assessments
    e. Development of facility IPC action plans
    f. Direct technical assistance for facility compliance with IPC standards and guidelines
    g. Development of IPC monitoring and evaluation systems
    h. Other, please specify ___________

5. Overall, how would you rate the quality of the collaboration between MTaPS and your health facility?
    a. Excellent
    b. Good
    c. Fair
    d. Poor

6. Do you think that the MTaPS approaches for collaboration meaningfully supported local ownership over IPC programs at your health facility?
    a. Yes, mostly supportive of local ownership
    b. Yes, sometimes supportive of local ownership
    c. No, rarely supportive of local ownership
    d. No, never supportive of local ownership

7. *(If q6 = a or b)* Please describe the three most important collaboration activities and how they fostered local ownership.
    a. _______

b. _______
c. _______

8. *(If q6 = c or d)* Please suggest three specific ways in which MTaPS approaches to collaboration could have better fostered local ownership.
   a. _______
   b. _______
   c. _______

Section 3. Approaches to MTaPS capacity strengthening with health facility stakeholders for Infection Prevention and Control (IPC)

9. Through what methods has MTaPS sought to strengthen the capacity of health facility staff? Select all that apply
   a. E-learning courses
   b. Specific just-in-time virtual trainings
   c. In-person training
   d. On-the-job monitoring and supervision
   e. Virtual mentoring (e.g., via WhatsApp, Zoom, etc.)
   f. Guidelines and toolkits
   g. Other (please specify) ____

10. Overall, how would you rate the quality of capacity strengthening between MTaPS and your health facility staff?
    a. Excellent
    b. Good
    c. Fair
    d. Poor

11. *(If q10 = a or b)* Please describe the three major elements of the MTaPS approach to capacity strengthening among health facility staff that you perceive as most impactful.
    a. _______
    b. _______
    c. _______

12. *(If q10 = c or d)* Please describe the three major areas lacking in the MTaPS approach to capacity strengthening among health facility staff.
    a. _______
    b. _______
    c. _______

13. Do you think that the MTaPS approach to capacity strengthening meaningfully supported local ownership over IPC programs at your health facility?
    a. Yes, mostly supportive of local ownership
    b. Yes, sometimes supportive of local ownership
    c. No, rarely supportive of local ownership
    d. No, never supportive of local ownership

Section 4. Compliance with Infection Prevention and Control (IPC) standards and guidelines before and during the COVID-19 outbreak and emergency response

14. Overall, in your health facility, how would you rate the level of compliance to IPC standards and guidelines prior to and during the COVID-19 pandemic? *(choices presented in a matrix)*
    a. Prior to the COVID-10 pandemic
        a. Excellent
        b. Good
        c. Fair
        d. Poor

    b. During the COVID-19 pandemic
        a. Excellent
        b. Good
        c. Fair
        d. Poor

15. In a few short sentences, please describe why you rated the level of compliance to IPC standards and guidelines before the COVID-19 pandemic this way.
    Open response

16. In a few short sentences, please describe why you rated the level of compliance to IPC standards and guidelines during the COVID-19 pandemic this way
    Open response

Section 5. COVID-19 pandemic response

17. Were there gaps in COVID-19 pandemic IPC response which were not adequately addressed by the prior (pre-Covid) MTaPS-supported GHSA IPC interventions at your health facility?
    a. Yes, many gaps
    b. Yes, some gaps
    c. Yes, few gaps
    d. No, no gaps

18. *(If q17 = a-c)* Please describe what you perceive as the three most substantial gaps in the COVID-19 IPC response that were not addressed by prior (pre-Covid) MTaPS-supported GHSA IPC programs.
    a. _______
    b. _______
    c. _______

19. To what extent do you agree with the following statement: MTaPS-supported GHSA pre-pandemic IPC program and capacity strengthening interventions enhanced my facility's response to the COVID-19 pandemic.
    a. Strongly agree
    b. Agree
    c. Disagree
    d. Strongly disagree

20. *(If q19 = a or b)* Please provide three main reasons why you believe the MTaPS-supported GHSA pre-pandemic IPC program and capacity strengthening interventions enhanced your facility's response to the COVID-19 pandemic.
    Open response

21. *(If q19 = b or c)* Please provide three main reasons why you believe the MTaPS-supported GHSA pre-pandemic IPC program and capacity strengthening interventions did not enhance your facility's response to the COVID-19 pandemic.
    Open response

22. Were the IPC systems that MTaPS helped to develop or strengthen adapted to respond to the COVID-19 pandemic in your facility?
    a. Yes, many adaptations were made
    b. Yes, some adaptations were made
    c. Yes, few adaptations were made
    d. No, no adaptations were made

Section 6. Summary 1uestions

23. Please tell us any other ways that MTaPS support impacted governance, capacity and institutionalized good IPC practices at MTaPS-supported facilities?
    Open response

24. In closing, please tell us any other ways, if at all, MTaPS support strengthened pandemic preparedness and response capacity the facility level?
    Open response

Section 6. Personal and contact information

25. Please list your position / job title
    Open response

26. What type of facility do you work in?
    a. Hospital
    b. Primary care- urban
    c. Primary care- rural
    d. Other (please specify) ________

27. How long have you been in this position?
    a. < 1 year
    b. 1 - <5 years
    c. 5 - <10 years
    d. 10+ years

28. Please select your primary country of work (*drop down options for all GHSA countries*)

29. In a few short sentences, describe your role in implementation of the GHSA COVID-19 response and/or prior IPC work
    Open response

30. May we contact you if we have further questions?
    a. No
    b. Yes

31. (*If q30= b)* What is your email address?

2. ***MTaPS national-level stakeholder survey***

Introduction and consent

Hello. You are being asked to kindly participate in this survey. This survey is commissioned by MSH-led USAID Medicines, Technologies, and Pharmaceutical Services (MTaPS) program and conducted by researchers at the Boston University School of Public Health. You have been identified because you are a key stakeholder in one or more of the programs supported by MTaPS that focuses on infection prevention control, antimicrobial resistance stewardship, or multisectoral collaboration on antimicrobial resistance (AMR) in your country. We believe you have valuable experience with and/or knowledge of these MTaPS-supported activities in your country before and during the COVID-19 pandemic.

We want to understand the collective impact of these programs and to generate lessons learned for pandemic preparedness. Could you please fill out this survey now? It should take about 20-30 minutes. The information you provide will have no impact on you and will be kept confidential. We will store your information in ways we think are secure. We will store electronic files in computer systems with password protection and encryption. However, we cannot guarantee complete confidentiality. Results will only be presented in aggregate. Completely anonymized data may be posted to a repository. You will not receive any direct benefits or compensation; results will help to make future programs better.

At the end of the survey, we will ask if we can follow up if we have additional questions. If you choose, you can input your contact information. If you have any questions about this survey, please contact [Boston University personnel, redacted]

Do you agree to participate?

c. No
d. Yes

If respondent answers yes, the survey continues (if no, it ends):

Welcome note

Thank you for agreeing to participate. Your responses will help us to understand strengths and weaknesses of the MTaPS capacity strengthening approach and make improvements to these programs in the future. Please answer to the best of your ability. We greatly appreciate your participation and perspectives!

Section 1. MTaPS-supported multisectoral coordination and Infection Prevention Control (IPC) programs and activities

32. With which MTaPS-supported programs have you been involved? Select all that apply.
    a. GHSA AMR package: Multisectoral and partner coordination for AMR and IPC
    b. GHSA AMR package: IPC strengthening at the national or sub-national level
    c. GHSA AMR package: IPC strengthening at the facility level
    d. MTaPS COVID-19 IPC package: Multisectoral and partner coordination for COVID-19 IPC at the national or subnational level
    e. MTaPS COVID-19 IPC package: COVID-19 IPC strengthening at the facility level

33. What kinds of activities have you been involved in? Select all that apply.
    a. Establishing or participating in governance structures (e.g. development of treatment guidelines, setting up task force, etc.)
    b. Establishing or strengthening the function of MSC-AMR body or its IPC technical working group (TWG)
    c. Advocating for funding initiatives
    d. Identifying and addressing national gaps in IPC policies/guidelines
    e. Building IPC capacity of healthcare providers and stakeholders
    f. Conducting IPC baseline and follow-up facility-level assessments
    g. Providing facility-level support
    h. Other (please specify) _________

Section 2. Approaches to MTaPS collaboration with government and national stakeholders for Infection Prevention and Control (IPC)

34. Through what methods has MTaPS collaborated with members of government and national stakeholders? Select all that apply.
    a. Supported national or sub-national IPC technical working groups (TWG)
    b. Supported to develop and/or update and/or disseminate national IPC guidelines
    c. Supported to establish infection control committees (ICCs) at one or more health systems levels
    d. Supported to advocate for mainstreaming IPC and/or WASH into planning and budgeting process
    e. Supported to assess national level IPC and WASH systems for human and animal health
    f. Supported to develop and/or implement action plans to address identified gaps at national or health facility levels
    g. Other (please specify) _________

35. Overall, how would you rate the quality of the collaboration between MTaPS and your government and national stakeholders?
    a. Excellent
    b. Good
    c. Fair
    d. Poor

36. *(If q4 = a or b)* Please describe the three major elements of the MTaPS approach that fostered collaboration among national stakeholders that you perceive as most impactful.
    a. ________
    b. ________
    c. ________

37. *(If q4 = c or d)* Please describe the three major areas lacking in the MTaPS approach to fostered collaboration among national stakeholders.
    a. ________
    b. ________
    c. ________

38. Do you think that the MTaPS approaches for collaboration meaningfully supported national ownership over IPC programs throughout the country?
    a. Yes, mostly supportive of national ownership
    b. Yes, sometimes supportive of national ownership

c. No, rarely supportive of national ownership
d. No, never supportive of national ownership

39. *(If q7 = a or b)* Please describe the three most important collaboration activities and how they fostered national ownership.
    a. ________
    b. ________
    c. ________

40. *(If q7 = c or d)* Please suggest three specific ways in which MTaPS approaches to collaboration could have better fostered national ownership.
    a. ________
    b. ________
    c. ________

Section 3. Approaches to MTaPS capacity strengthening with government and national stakeholders for Infection Prevention and Control (IPC)

41. Through what methods has MTaPS sought to strengthen the capacity of government and national stakeholders? Select all that apply.
    a. E-learning courses
    b. Specific just-in-time virtual trainings
    c. In-person training
    d. On-the-job monitoring and supervision
    e. Virtual mentoring (e.g., via WhatsApp, Zoom, etc.)
    f. Guidelines and toolkits
    g. Other (please specify) _____

42. Overall, how would you rate the quality of capacity strengthening between USAID MTaPS and your government and national stakeholders?
    a. Excellent
    b. Good
    c. Fair
    d. Poor

43. *(If q11 = a or b)* Please describe the three major elements of the MTaPS approach to capacity strengthening among national stakeholders that you perceive as most impactful.
    a. ________
    b. ________
    c. ________

44. *(If q11 = c or d)* Please describe the three major areas lacking in the MTaPS approach to capacity strengthening among national stakeholders.
    a. ________
    b. ________
    c. ________

45. Do you think that the MTaPS approaches to capacity strengthening meaningfully supported national ownership over IPC programs?
    a. Yes, mostly supportive of national ownership
    b. Yes, sometimes supportive of national ownership

c. No, rarely supportive of national ownership
d. No, never supportive of national ownership

46. *(If q14 = a or b)* Please describe the three most important capacity strengthening activities and how they fostered national ownership.
    a. ________
    b. ________
    c. ________

47. *(If q14 = c or d)* Please suggest three specific ways in which MTaPS approaches to capacity strengthening could have better fostered national ownership.
    a. ________
    b. ________
    c. ________

Section 4. Compliance with Infection Control and Prevention (IPC) standards and guidelines before and during the COVID-19 outbreak and emergency response

48. Overall, in MTaPS-supported facilities in your country, how would you rate the level of compliance to IPC standards and guidelines prior to the start of the COVID-19 pandemic compared with during the COVID-19 pandemic? (*choices presented in a matric)*
    a. Before the COVID-19 pandemic
        i. Excellent
        ii. Good
        iii. Fair
        iv. Poor
    b. During the COVID-19 pandemic
        i. Excellent
        ii. Good
        iii. Fair
        iv. Poor

49. In a few short sentences, please describe why you rated the level of compliance before the COVID-19 pandemic this way.
    Open response

50. In a few short sentences, please describe why you rated the level of compliance during the COVID-19 pandemic this way.
    Open response

51. Please describe three major factors that made complying to IPC standards and guidelines before the COVID-19 pandemic difficult.
    a. ________
    b. ________
    c. ________

52. Please describe three major factors that made complying to IPC standards and guidelines during the COVID-19 pandemic difficult.
    a. ________
    b. ________
    c. ________

53. Please describe three major factors that helped to make complying to IPC standards and guidelines before the COVID-19 pandemic easier.
    a. ________
    b. ________
    c. ________

54. Please describe three major factors that helped to make complying to IPC standards and guidelines during the COVID-19 pandemic easier.
    a. ________
    b. ________
    c. ________

Section 5. COVID-19 Pandemic Response

55. Overall, how would you rate MTaPS support for multisectoral coordination activities on AMR in relation to the COVID-19 response?
    a. Excellent
    b. Good
    c. Fair
    d. Poor

56. In a few short sentences, please describe why you rated the level of multisectoral coordination activities on AMR in relation to the COVID-19 response this way.
    Open response

57. Overall, in MTaPS-supported facilities in your country, were there gaps in COVID-19 pandemic IPC response which were not adequately addressed by GHSA IPC interventions?
    a. Yes, many gaps
    b. Yes, some gaps
    c. Yes, few gaps
    d. No, no gaps

58. *(If q26 = a-c)* Please describe what you perceive as the three most substantial gaps in the COVID-19 IPC response that were not addressed by prior MTaPS supported GHSA IPC programs.
    a. ________
    b. ________
    c. ________

59. To what extent do you agree with the following statement: MTaPS-supported GHSA pre-pandemic IPC program and capacity strengthening interventions enhanced my country's response to the COVID-19 pandemic.
    a. Strongly agree
    b. Agree
    c. Disagree
    d. Strongly disagree

60. *(If q28 = a or b)* Please provide three main reasons why you believe the MTaPS-supported GHSA pre-pandemic IPC program and capacity strengthening interventions enhanced your country's response to the COVID-19 pandemic

a. ________
b. ________
c. ________

61. *(If q28 = c or d)* Please provide three main reasons why you believe the MTaPS-supported GHSA pre-pandemic IPC program and capacity strengthening interventions did not enhance your country's response to the COVID-19 pandemic
    a. ________
    b. ________
    c. ________

62. Did the IPC systems that MTaPS helped develop or strengthen need to be adapted to respond to the COVID-19 pandemic in your country?
    a. Yes, many adaptations were made
    b. Yes, some adaptations were made
    c. Yes, few adaptations were made
    d. No, no adaptations were made

63. *(If q31 = a-c)* In a few short sentences, please explain how your country adapted pre-pandemic IPC systems to address the COVID-19 pandemic.
    Open response

64. Please list three lessons you learned in your COVID-19 response that will help your country continue to strengthen IPC program capacity going forward.
    a. ________
    b. ________
    c. ________

65. Were non-MTaPS-supported approaches to improving IPC practices implemented before or during the COVID-19 pandemic?
    a. Yes, before
    b. Yes, during
    c. Yes, both before and during
    d. No

66. *(If q34 = a-c)* Please explain how non-MTaPS-supported approaches to IPC were implemented before or during the COVID-19 pandemic
    Open response

67. *(If q34 = a-c)* Did non-MTaPS-supported approaches contribute to observed outcomes in IPC compliance and the COVID-19 response? If so, please explain how.

Section 6. Summary questions

68. Please tell us any other ways that MTaPS support impacted governance, capacity and institutionalized good IPC practices at MTaPS-supported facilities?
    Open response

69. In closing, please tell us any other ways, if at all, MTaPS support strengthened pandemic preparedness and response capacity at both the facility and national levels?
    Open response

Section 7. Personal and contact information

70. Please list your position / job title
    Open response

71. How long have you been in this position?
    a. < 1 year
    b. 1 - <5 years
    c. 5 - <10 years
    d. 10+ years

72. Please select your primary country of work (*drop down options for all GHSA countries*)

73. In a few short sentences, describe your role in implementation of the GHSA COVID-19 response and/or prior IPC work
    Open response

74. May we contact you if we have further questions?
    a. No
    b. Yes

75. (*If q29 = b)* What is your email address?

3. ***MTaPS-Contracted Country Implementers Survey***

Introduction and consent

Hello. You are being asked to kindly participate in this survey. This survey is commissioned by MSH-led USAID Medicines, Technologies, and Pharmaceutical Services (MTaPS) program and conducted by researchers at the Boston University School of Public Health. You have been identified because you are a key stakeholder in one or more of the programs supported by MTaPS that focuses on infection prevention control, antimicrobial resistance stewardship, or multisectoral collaboration on antimicrobial resistance (AMR) in your country. We believe you have valuable experience with and/or knowledge of USAID MTaPS-supported IPC activities at national, regional, and or local level before and during the COVID-19 pandemic.

We want to understand the collective impact of these programs and to generate lessons learned for pandemic preparedness. Could you please fill out this survey now? It should take about 15-20 minutes. The information you provide will have no impact on you and will be kept confidential. We will store your information in ways we think are secure. We will store electronic files in computer systems with password protection and encryption. However, we cannot guarantee complete confidentiality. Results will only be presented in aggregate. Completely anonymized data may be posted to a repository. You will not receive any direct benefits or compensation; results will help to make future programs better.

At the end of the survey, we will ask if we can follow up if we have additional questions. If you choose, you can input your contact information. If you have any questions about this survey, please contact [Boston University personnel, redacted]

Do you agree to participate?

a. No
e. Yes

If respondent answers yes, the survey continues (if no, it ends):

Welcome note

Thank you for agreeing to participate. Your responses will help us to understand strengths and weaknesses of the MTaPS capacity strengthening approach and make improvements to these programs in the future. Please answer to the best of your ability. We greatly appreciate your participation and perspectives!

Section 1. Compliance with Infection Prevention and Control (IPC) standards and guidelines

1. Overall, in MTaPS-supported health facilities in your country, how would you rate the level of compliance to COVID-19 IPC standards and guidelines during the COVID response phase?
    a. Excellent
    b. Good
    c. Fair
    d. Poor

2. In a few short sentences, please describe why you rated level of compliance this way.
    Open response

3. Please describe three major factors that made complying to IPC standards and guidelines <u>difficult.</u>
    a. _______
    b. _______
    c. _______

4. Please describe three major factors that helped to make complying to IPC standards and guidelines <u>easier</u>.
    a. _______
    b. _______
    c. _______

Section 2. Gaps in the COVID-19 response

5. Overall, in MTaPS-supported health facilities in your country, were there gaps in COVID-19 pandemic IPC response which were not adequately addressed by GHSA or IPC interventions?
    a. Yes, many gaps
    b. Yes, some gaps
    c. Yes, few gaps
    d. No, no gaps

6. *(If q5 = a-c)* Please describe what you perceive as the three most substantial gaps in the COVID-19 IPC response that were not addressed by the GHSA IPC interventions.

a. ________
b. ________
c. ________

Section 3. Approaches to MTaPS collaboration and capacity strengthening with government and national stakeholders

7. Overall, how would you rate the quality of the collaboration between MTaPS and your government and national stakeholders?
    a. Excellent
    b. Good
    c. Fair
    d. Poor

8. Please describe the elements of MTaPS' approach to foster collaboration among national stakeholders that you perceive as most impactful (e.g., collaboration between Ministry of Health and other partners) to implement GHSA/IPC standards and guidelines in your country.
    Open response

9. Overall, how would you rate the quality of capacity strengthening between MTaPS and your government and national stakeholders?
    a. Excellent
    b. Good
    c. Fair
    d. Poor

10. Which elements of MTaPS approach to capacity strengthening among national stakeholders do you perceive as most impactful in your country? Select all that apply.
    a. E-learning courses
    b. Specific just-in-time virtual trainings
    c. In-person training
    d. On-the-job monitoring and supervision
    e. Virtual mentoring (e.g., via WhatsApp, Zoom, etc.)
    f. Guidelines and toolkits
    g. Other (please specify) _____

11. Do you think that the MTaPS approaches for collaboration and capacity strengthening meaningfully supported national ownership over implementing the GHSA and IPC standards and guidelines?
    a. Yes, mostly supportive of national ownership
    b. Yes, sometimes supportive of national ownership
    c. No, only rarely supportive of national ownership
    d. No, never supportive of national ownership

12. *(If q11=a or b)* Please describe the supportive activities and how they fostered national ownership.
    Open response

13. (*If q11= c or d*) Please suggest three specific ways in which MTaPS approaches to collaboration and capacity strengthening could have better fostered national ownership.
    a. ________

    b. _______
    c. _______

Section 4. Global Health Security Agenda (GHSA) Infection Prevention Control (IPC) and Multisectoral Coordination on AMR: pandemic preparedness and response

14. Overall, in MTaPS-supported facilities in your country, how would you rate the level of compliance to IPC standards and guidelines prior to the start of the COVID-19 pandemic?
    a. Excellent
    b. Good
    c. Fair
    d. Poor

15. In a few short sentences, please describe why you rated the level of compliance to IPC standards and guidelines before the COVID-19 pandemic this way.
    Open response

16. Did GHSA IPC and multisectoral coordination on AMR systems that MTaPS helped strengthen have to be adapted to address the COVID-19 pandemic in your country?
    a. Yes, many adaptations were made
    b. Yes, some adaptations were made
    c. Yes, few adaptations were made
    d. No, no adaptations were made

17. (*If q16=a-c*) In a few short sentences, please explain how your country adapted pre-pandemic GHSA IPC systems to address the COVID-19 pandemic
    Open response

18. (*If q16=a-c*) In a few short sentences, please explain how your country adapted pre-pandemic GHSA work relating to multisectoral coordination on AMR to address the COVID-19
    Open response

19. Overall, how would you rate MTaPS support for multisectoral coordination activities on AMR in relation to the COVID-19 response?
    a. Excellent
    b. Good
    c. Fair
    d. Poor

20. In a few short sentences, please describe why you rated the level of multisectoral coordination activities on AMR in relation to the COVID-19 response this way.
    Open response

21. List 2 lessons you learned in your COVID-19 response that will help your country continue to strengthen IPC program capacity going forward.
    a. _______
    b. _______

22. List 2 lessons you learned in your COVID-19 response that will help your country continue to strengthen multisectoral coordination on AMR capacity going forward.
    a. _______

b. ________

Section 5. Summary questions

23. Please tell us any other ways that MTaPS support impacted governance, capacity and institutionalized good IPC practices at MTaPS-supported facilities?
    Open response

24. In closing, please tell us any other ways, if at all, MTaPS support strengthened pandemic preparedness and response capacity at both the facility and national levels?
    Open response

Section 6. Personal and Contact Information

25. Please list your position / job title
    Open response

26. How long have you been in this position?
    a. < 1 year
    b. 1 - <5 years
    c. 5 - <10 years
    d. 10+ years

27. Please select your primary country of work (*drop down options for all GHSA countries*)

28. In a few short sentences, describe your role in implementation of the GHSA COVID-19 response and/or prior IPC work
    Open response

29. May we contact you if we have further questions?
    a. No
    b. Yes

30. (*If q29 = b)* What is your email address?

Figure S1. MSC-AMR-related activities and outputs

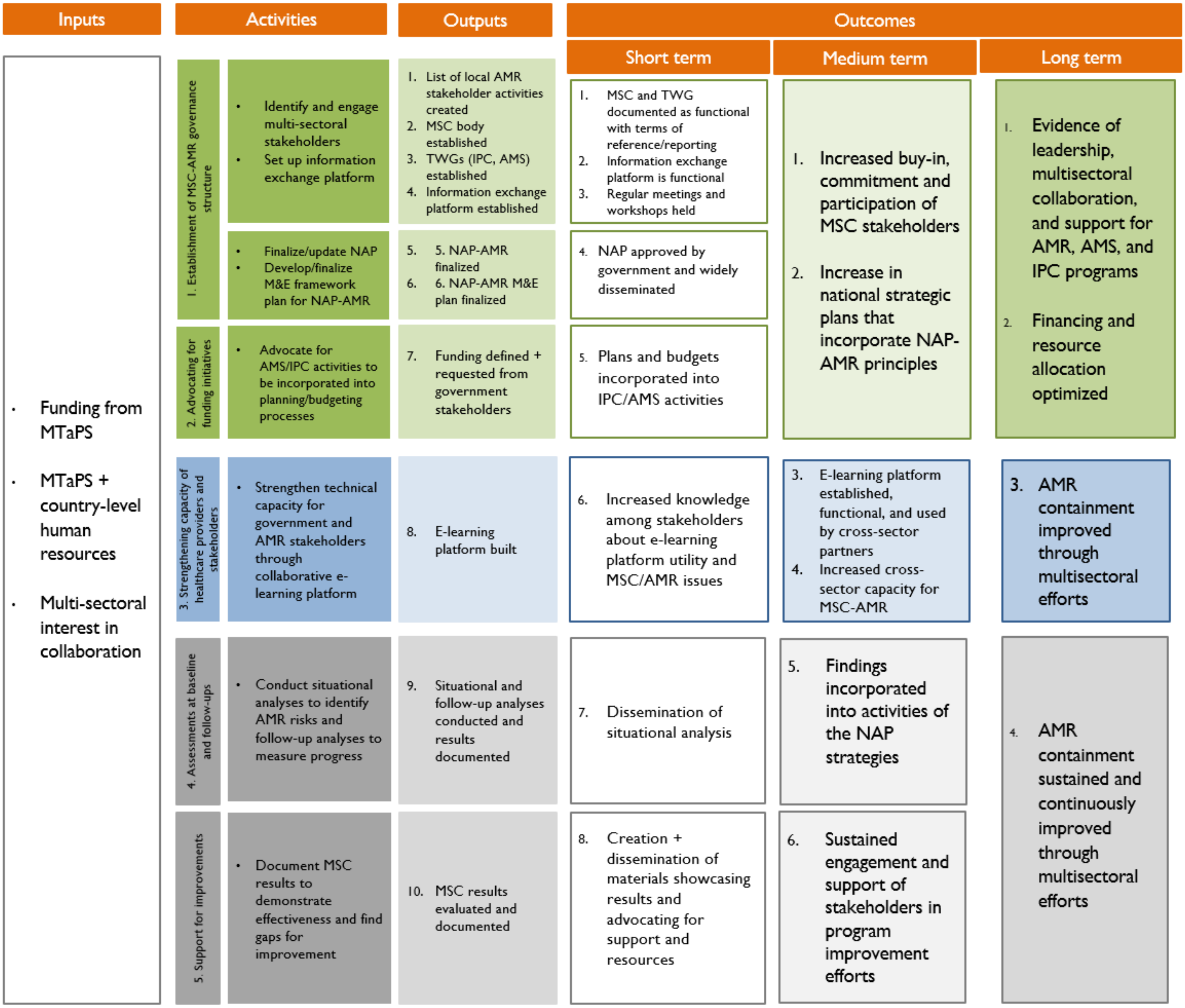

Abbreviations: MSC: multi-sectoral coordination; **AMR**: antimicrobial resistance; **TWG**: technical working group; **AMS**: antimicrobial stewardship; **IPC**: infection prevention and control; **NAP:** national action plan; **e-learning**: electronic learning; **M&E**: monitoring and evaluation

Legend. Figure S1 depicts a logic model for MTaPS Multi-Sectoral Coordination on Antimicrobial Resistance (MSC-AMR) program. Inputs represent the resources and efforts needed for the program, and activities represent planned aspects of the program. Outcomes are divided into short, medium, and long-term outcomes. The logic behind the program is that intervention activities will lead to outcomes. Logic models were derived from detailed program implementation plans (unpublished).

Figure S2. IPC-related activities and outputs

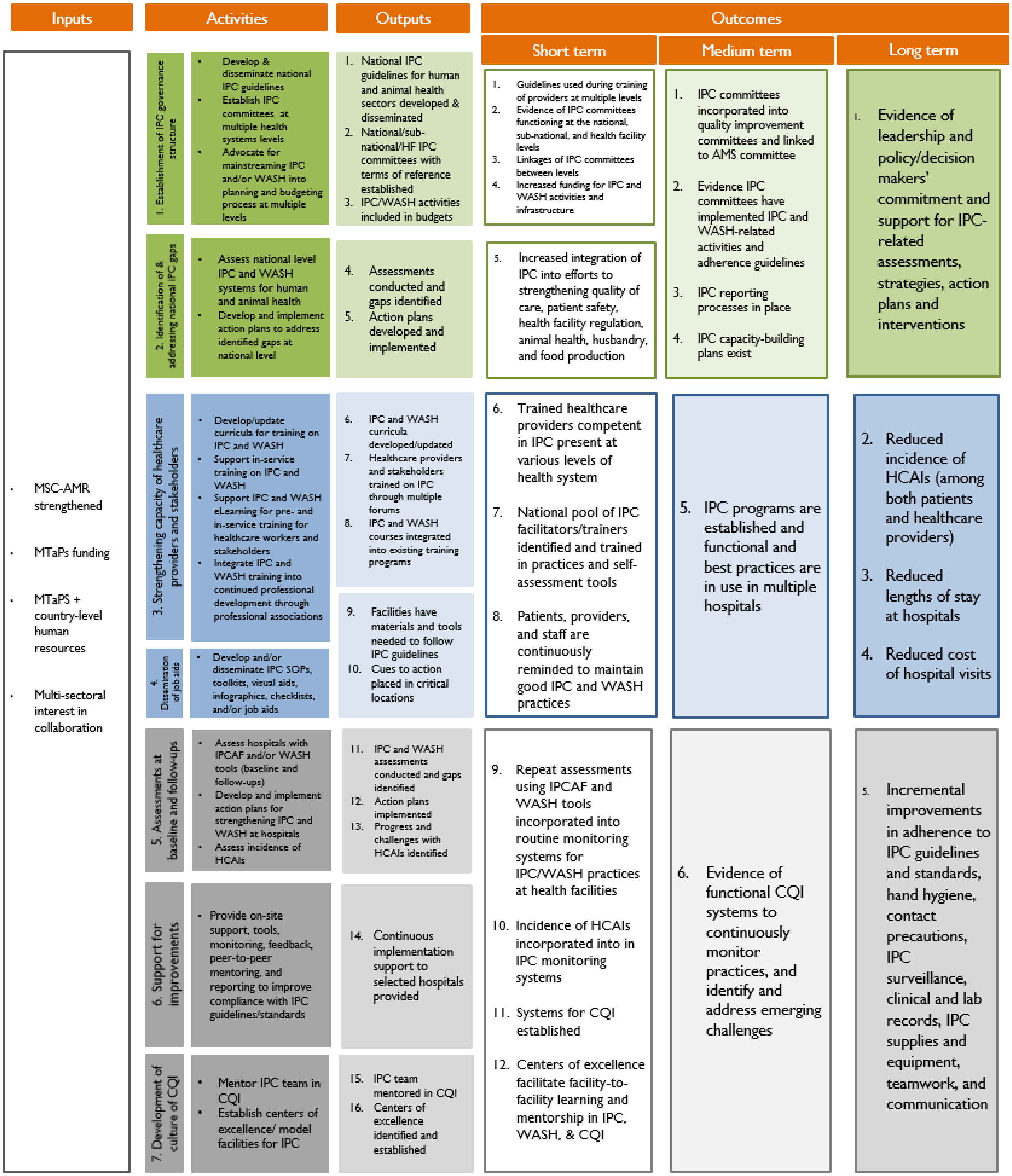

Abbreviations: IPC: infection prevention and control; **ICC:** infection control committee; **HCAIs:** health care-associated infections; **CQI:** continuous quality improvement

Legend. Figure S2 depicts a logic model for MTaPS Infection Prevention and Control (IPC) program. Inputs represent the resources and efforts needed for the program, and activities represent planned aspects of the program. Outcomes are divided into short, medium, and long-term outcomes. The logic behind the program is that intervention activities will lead to outcomes. Logic models were derived from detailed program implementation plans (unpublished).
